# Giant stone in a urinary bladder diverticulum in a 69-year-old male: a case report

**DOI:** 10.11604/pamj.2023.45.181.38723

**Published:** 2023-08-25

**Authors:** Syarif Syarif, Abdul Azis, Muhammad Ilham Fauzan Patimura, Muhammad Zulharyahya Dandy Asmara Putra, Ade Nusraya, Ahmad Shafwan Natsir

**Affiliations:** 1 Department of Surgery, Faculty of Medicine Hasanuddin University, Makassar, South Sulawesi, Indonesia,; 2 General Practicioner, Siloam Hospital, Makassar, South Sulawesi, Indonesia

**Keywords:** Bladder stone, bladder diverticulum, bladder neck stenosis, transurethral resection, case report

## Abstract

The stone formation could occur due to urine stasis in the bladder diverticulum. However, the stones are usually smaller in size and can pass spontaneously. However, a giant stone inside vesical diverticulum is considered a rare entity. We report a 69-year-old male, with a two-year history of lower urinary tract symptoms along with a recurrence of urinary tract infection. An abdominal computed tomography scan revealed the presence of a giant bladder diverticulum and a large bladder stone. The patient underwent a transurethral bladder neck incision followed by diverticulectomy with stone extraction. The diverticulum size measures 6x4x3.8 cm and diverticulum stone size of 4x3x3 cm. Fortunately, the patient recovered well after the operation. In conclusion, giant stones inside large vesical diverticulum are a rare occurrence and should be considered in patients with lower urinary tract symptoms. Early diagnosis and optimal management of the obstruction are the principles to prevent long-term complications.

## Introduction

The Bladder diverticulum is a herniation of the bladder mucosa and submucosa through the muscular wall of the bladder, resulting in a loss of bladder wall contractility and urine stasis in the diverticulum [1]. A secondary Bladder diverticulum is known to be caused by a prolonged increase of intra-vesical pressure caused by lower urinary tract obstruction [2]. Today, transurethral resection of the prostate (TURP) is the most common modality for treating Benign prostate hyperplasia. However, TUR-P is also unseparated from the possibility of complications of bladder neck stenosis. Based on the previous study by Lee YH, 12.3% of patients who underwent TURP had bladder neck stenosis [3]. The stone formation could occur due to urine stasis in the diverticulum. However, stones are usually smaller and can pass spontaneously through the diverticulum lumen. A giant stone inside the vesical diverticulum is considered a very rare incident. Herein we report a case of a 69-year-old male patient with giant bladder diverticulum accompanied by an oval-shaped intra-diverticular urinary bladder stone. Through this case and a review of the literature, we will discuss the epidemiological, diagnostic, and therapeutic aspects.

## Patient and observation

**Patient information:** a 69-year-old male presents with a two-year history of difficulty to hold urine once the urge initiates associated with daytime urinary frequency, poor urinary stream, sensation of incomplete emptying, occasional hematuria, and recurrence of urinary tract infection. The complaints have been worse in the last six months. Previously, the patient consumed an α-adrenergic blocker (Tamsulosin 0,4 mg daily) for the last four months without improvement. The patient underwent transurethral resection of the prostate (TURP) 10 years ago. The patient was also diagnosed with hypertension ten years ago and consumed calcium channel blockers (Amlodipine 10 mg daily) regularly. No history of other chronic illnesses. No significant disease in his family history.

**Clinical findings:** blood pressure 148/90 mmHg, respiratory rate 16x/min, heart rate 90x/min, and axial temperature 38°C. The thorax, abdominal and neurological examination revealed no abnormality. A digital rectal examination revealed a normal anal tone, normal perianal sensation, and a small, smooth prostate.

**Figure 1:**
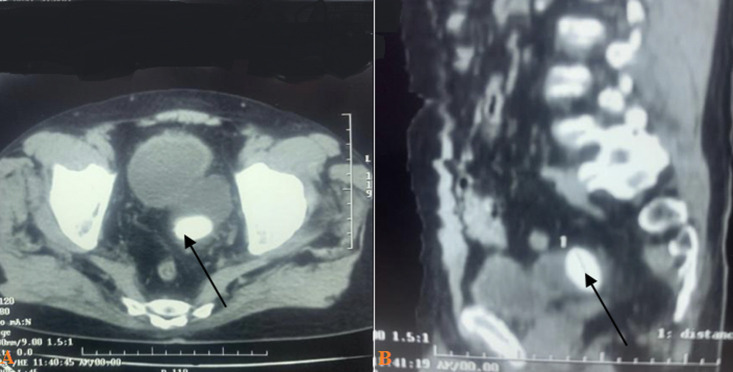
A) axial cross-section of non-contrast abdominal computerizing tomography-scan showing a bladder diverticulum with vesicolithiasis inside; B) sagittal cross-section of non-contrast abdominal computerizing tomography-scan

**Diagnostic assessment:** non-contrast abdominal computed tomography (CT) scan revealed a severe bladder wall thickness, bladder diverticulum in the left posterior wall of the bladder with a size of 6x4x3.8 cm, oval-shaped intra-diverticular stone size of 3x2x2.9 cm, and prostate enlarged with a volume 34 ml (Figure 1A, Figure 1B). A hematology assessment showed leukocytosis (16.60 u/L). Serum creatinine and blood urea nitrogen (BUN) concentrations were within normal limits at a value of 1.05 mg/dL and 35 mg/dL. Prostate specific antigen (PSA) at a value of 7 ng/mL, urinalysis results contained leukocytes esterase and more than 20 white blood cells. Urine culture grew *Escherichia coli* and *Staphylococcus saprophyticus* and both organisms were sensitive to ceftriaxone.

**Figure 2:**
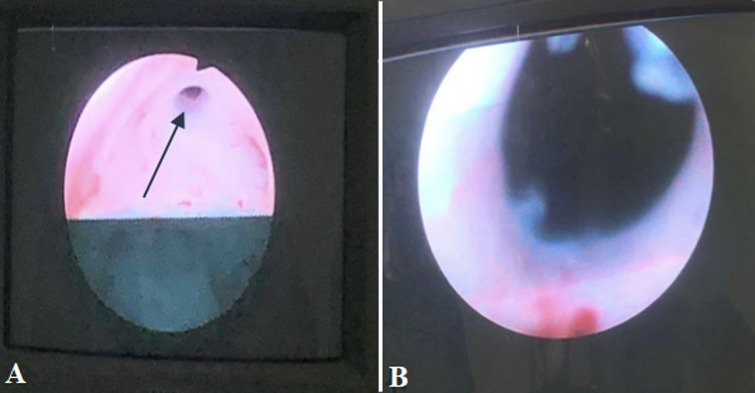
A) the urethra lumen before the urethrotomy and transurethral resection; B) after the urethrotomy and transurethral resection, the lumen looks wider

**Figure 3:**
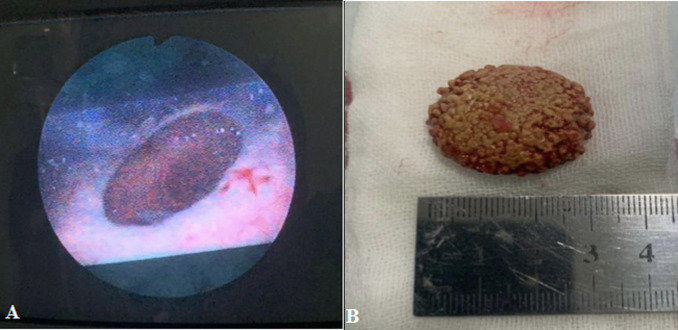
A) oval-shaped vesicolithiasis seen from the mouth of the diverticulum; B) the imaging of the diverticular stone after its removal from the bladder diverticulum

**Therapeutic intervention:** the patient was given ceftriaxone 1g intravenously 2 hours before the surgery. After receiving spinal anesthesia, the patient was positioned in the lithotomy position. Initially, to confirm the cause of the lower urinary tract obstruction, urethroscopy was performed with a 12 Fr rigid urethroscope, and a warm saline solution was used for irrigation. After the urethroscope entered the urethra, there was a lumen narrowing inside the bladder neck, which inhibited the urethroscope from entering the bladder (Figure 2 A). Then, the urethrotomy procedure and transurethral resection were performed to scrape the wall of the bladder neck in order to widen the lumen and remove the obstruction (Figure 2 B). After the camera entered the bladder, there was severe trabeculation throughout the bladder wall and a round-shaped defect with a diameter of 1.5 cm located just above the trigone on the posterior wall of the bladder with a distance of 1.5 cm from the left ureteral orifice and 2 cm from the right one (Figure 3 A) and also there was a giant stone inside the diverticulum. After the urethocystocopy was completed, a 14 Fr Foley Catheter was inserted and then prepared to perform an open cystostomy. The vertical suprapubic incision was made with a length of ±10 cm. After the bladder opened, a diverticulectomy was performed to remove the abnormal poach. A circular incision was made on the mouth of the diverticulum to separate the bladder mucosa from the poach and facilitate the removal of the diverticulum stone. An oval-shaped stone with an irregular surface in size of 4x3x3 cm, with a weight of 160 gr was obtained (Figure 3 B). The bladder wall reconnected by suturing the muscles and mucosa separately. The total operative time was about 120 minutes, and the estimated blood loss was 300 ml.

**Follow-up and outcomes:** the patient was sent to the intensive care unit (ICU) after the surgery and recovered well before subsequently being referred back to the urology ward. Removal of the drain was done on the third postoperative day and the ureteral catheter was removed on the seventh postoperative day. Two weeks after surgery, the patient came to the urology polyclinic for follow-up control. The patient complains about his postoperative pain. The complaints of storage and voiding Lower urinary tract symptoms (LUTS) were improved after the surgery. Ultrasonography was performed to confirm the improvement and to check if there were any residual complications after the surgery, and no abnormality was found. Antibiotics (Cefixime 200 mg twice a day) and analgetic (Meloxicam 15 mg daily) were described for his pain.

**Patient´s perspective:** the patient was satisfied with the result of the surgery. The complaints of storage and voidance LUTS have improved after the surgery.

**Informed consent:** written informed consent was obtained from the patient for participation in our study.

## Discussion

A bladder diverticulum is a rare urology entity in both the pediatric and adult populations. It is classified as congenital and acquired. The more common type is the secondary (acquired) diverticulum, which is known to be the result of increased intravesical pressure caused by lower urinary tract obstruction, with up to 80% being attributed to benign prostatic hypertrophy (BPH) [1]. In this case report, the patient has an acquired bladder diverticulum, it was caused by prolonged obstruction on the lower part, which increases the pressure in the urinary bladder, subsequently making the muscles in the bladder wall work harder to compensate for the increased pressure, it was also proven by the cystoscopy imaging of the patient which showed there are severe trabeculation throughout the layer of the urinary bladder. Later, causing bladder wall weakness and herniation, which form the diverticulum. Lower urinary tract obstruction in our case was caused by bladder neck stenosis, which was found during the urethroscopy. Several risk factors could cause bladder neck stenosis, including small prostate volume, postoperative urinary tract infection, unsuitable resectoscope, history of prostatitis, extensive resection of the bladder neck, long surgical time, and re-catheterization after surgery [4]. However, one of the most reported risk factors is a history of transurethral resection of the prostate (TURP) treatment. Based on a previous study by Anton Grechenkov in 2018, 20 out of 402 (4.97%) patients who underwent TURP had bladder neck stenosis (BNS) [4], another study by Puppo also reported two years after conventional TURP resulted in a high BNS rate of 12.3% [3]. Over-resection of the bladder neck can lead to fibrosis or scarring of the bladder neck, which later leads to bladder neck contraction [5]. This study also matches our case report, where the patients had a history of undergoing TURP ten years ago. Bladder diverticulum does not often present a distinct symptom and is most commonly detected accidentally while investigating another unrelated disease which leads to a delay in diagnosis [6]. Urinary tract infection secondary to post-void residual is the most common manifestation which is also found in the patient [6]. However, the case was also accompanied by bladder neck stenosis, so there is also a variety of symptoms including voiding symptoms, storage symptoms, or a combination of both [3].

The diagnosis was based on imaging, including ultrasonography, retrograde urethrocystography, computed tomography (CT) scan, and a cystoscopy [7]. CT scan of the urinary tract is recommended choice to precisely measure the volume of the diverticulum, to show a clearer picture of a possible impact on the upper urinary tract, and to specify the close relationship between the posterior surface of the diverticulum and the neighboring organs (rectum, and homolateral ureter) [7]. Cystoscopy specifies the dimensions of the neck of the diverticulum and its position in relation to the ureteral meatus. In some cases, a very wide neck can be crossed by the cystoscope, which allows visualization of the diverticulum wall and identification of a possible intra-diverticular ureteral orifice [7]. In this case, ultrasonography, CT scan, and urethro-cystoscopy were also performed to precisely diagnose. For the treatment of the secondary bladder diverticulum, the primary cause should be treated first to prevent it from recurring. There are various modalities used to treat bladder neck stenosis, such as urethral dilation, endoscopic incision, urethral stent, and reconstructive surgery [3]. Bladder neck contracture incision can be performed with a variety of techniques, including cold-knife, electrocautery, laser, hot-knife, and loop resection [3]. The combination of endoscopic balloon dilation with incision appears to offer promising results in the management of BNS [3]. Proven after a year, the majority of patients (72%) required only one BNS procedure which is 12% higher compared to endoscopic incision alone [3]. Observation is usually advised in asymptomatic patients. Surgical management via diverticulectomy is warranted whenever the patient suffers from recurrent UTIs, bladder stones, complicated vesicoureteral reflux, voiding dysfunction, and urinary retention [8]. Extraperitoneal or intraperitoneal approaches to the open abdomen are both possible. For the common diverticulum, a transvesical technique can be utilized, in which the anterior portion of the bladder wall is opened. Then the diverticulum is everted and circumcised, and then the bladder is closed [6]. In this case, diverticulectomy was performed using a transvesical approach. This method needs to be done meticulously. Otherwise, it will lead to vesicoureteral reflux, which often occurs in cases of bladder diverticulum [9]. Stone formation is one of the complications that may occur in the bladder diverticulum due to urine stasis [10]. However, the stones are usually small and can pass spontaneously through the diverticulum lumen to the urinary bladder. In this case, there was a stone measuring 4x3x3 cm with a weight of 160 gr in the bladder diverticulum which was quietly rare found in cases of bladder diverticulum.

## Conclusion

Giant stones inside large vesical diverticulum are a rare occurrence and should be considered in patients with lower urinary tract symptoms. Early diagnosis and optimal management of the obstruction are the principles to prevent long-term complications.
